# Portable devices in ophthalmology

**DOI:** 10.5935/0004-2749.20200111

**Published:** 2024-02-11

**Authors:** Daniel Araújo Ferraz, Dawn A. Sim, Rubens Belfort Junior, Victor Koh

**Affiliations:** 1 Vision Institute, São Paulo, SP, Brazil; 2 NIHR Biomedical Research Centre for Ophthalmology, Moorfields Eye Hospital, NHS Foundation Trust and UCL Institute of Ophthalmology, London, United Kingdom; 3 Department of Ophthalmology, National University of Singapore, Singapore

Dear Editor,

We have read, with strong enthusiasm, the *ABO* editorial by Malerbi^(1)^ that illustrated diabetic retinopathy screening during COVID-19 pandemic. While there is a great interest in understanding the program’s potential, we noticed some additional points to this, which have been discussed herein.

Telemedicine is currently limited by several challenges, namely, a safe and effective implementation model, human resources utilization, suitability of diagnostic equipment, stakeholders’ adoption, clinical data management, and medico-legal implications. These problems are applicable worldwide, and the current teleophthalmology models are based on two central systems of operation. The first system deploys permanent satellite centers managed by trained operators, such as optometrists. This system has been adopted in the United Kingdom at the Moorfields Eye Hospital in London in the form of a cloud-based referral platform. This system was created to address the issue of unnecessary referrals to specialists as well as to create a direct communication channel among community optometrists or doctors, with high-quality diagnostic tests alongside patient clinical information to be reviewed by an ophthalmologist. It has also enabled clinical services to be directed appropriately to patients requiring urgent ophthalmic intervention, as 54% of the referrals have been found to be unnecessary^(2)^. The second system uses a mobile teleophthalmology system that has been adopted in India, wherein it can travel to the rural areas where transportation and accessibility to eye care are suboptimal. This process requires a coordinated approach, starting from identifying suitable sites, increasing awareness/publicity, conducting population census, shipping the necessary equipment/team, and conducting teleconsultation and post-consultation follow-up. A typical setup in this system may consist of a group of trained optometrists and equipment, including autorefractor, slit lamp, tonometry, and nonmydriatic fundus camera. Frequently, for more complex eye conditions, video conferencing with an ophthalmologist is possible, which provides pointof-care triaging and the determination of follow-up care^(3)^. Moreover, in both the cases, the patient would has to travel to the ophthalmic site of examination at a certain time point, if not at present. One of the significant considerations for both the systems discussed above is that the diagnostic equipment deployed is bulky, expensive, and difficult to operate, with limiting scalability and accessibility. Both these systems however circumvent the need for an on-site ophthalmologist, which is a requirement of the teleophthalmology systems, although the present challenge is the lack of a suitable diagnostic ophthalmic equipment. In fact, there is an unmet need for a new breed of devices designed to be community-friendly, which includes the factors of portability, ease-of-use, and affordability. Ideally, these devices would be used by patients. Thus, new healthcare models and technologies need to be developed to meet the increasing demands of rapidly aging patient populations.

Presently, there are some commercially available portable diagnostic devices available in the ophthalmic field, such as fundus cameras, auto-refractors, slit lamps, and tonometry devices, for example, the Spot^®^ Vision Screener autorefractor, The Reichert^®^ TONO-PEN^®^ XL Tonometer, and Keeler PSL One Portable Slit Lamp. For the retinal examination, several portable cameras are presently available that can be connected for transferring images to specialists and reading centers. Favorable, several of these aspects are currently being consigned by industry and investors, which support the acquisition of color fundus photographs using smartphones with a condensing lens or an ophthalmoscopy adaptor such as the Portable Eye Examination Kit (PEEK), D-Eye Device, and Ocular Cellscope. The advantages of these systems include portable technology that enables high-quality image capturing to assess age-related macular degeneration and diabetic retinopathy (DR)^(4)^.

Portable devices play a significant role in monitoring and managing healthcare for a wide range of patients. A large-scale spectrum of portable medical devices is already available in the market, while various next-gen devices are still under development. These devices are popularly being used in ever-increasing applications such as those used for cardiac, respiratory, and wellness concerns, including the following: apnea monitors, continuous positive airway pressure machines, electrocardiogram monitors, blood glucose meters, insulin infusion pumps, blood pressure monitors, pulse oximeters, and ultrasound devices. The use of portable medical device is rapidly expanding since the advancements in the development of wireless technologies have enhanced home low-cost sensor technologies, low power consumption, repeatability, and reliability.

The new concept of miniaturized portable devices in ophthalmology is based on increasing accessibility, affordability, and integrability. The COVID-19 pandemic is believed to act a catalyst in promoting the acceptance and adoption of these devices to spur the development of new decentralized chronic eye care models complementing the pre-existing system. These devices do not just provide continuous and non-invasive monitoring of health parameters, but, through connectivity, also offer real-time updates to the healthcare workers. Portable devices possess the potential to revolutionize eye care, making high-quality services sustainable and accessible to everyone.

## References

[1] Malerbi FK, Morales PHA, Regatieri CVS. (2020). Diabetic retinopathy screening and the COVID-19 pandemic in
Brazil. Arq Bras Oftalmol.

[2] Sim DA, Mitry D, Alexander P, Mapani A, Goverdhan S, Aslam T (2016). The evolution of teleophthalmology programs in the united
kingdom: beyond diabetic retinopathy screening. J Diabetes Sci Technol.

[3] Bai VT, Murali V, Kim R, Srivatsa SK. (2007). Teleophthalmology-based rural eye care in India. Telemed J Health.

[4] Wintergerst MW, Mishra DK, Hartmann L, Shah P, Konana VK, Sagar P (2020). Diabetic retinopathy screening using smartphone-based fundus
imaging in Indian. Ophthalmology.

