# Supplementary material for: Portable devices in ophthalmology
Source: Arq Bras Oftalmol. 2024 Feb 11;83(6):566–7. doi: 10.5935/0004-2749.20200111 (PMC12289163; doi:10.5935/0004-2749.20200111)
Supplement: Translation

# Translation: Reply: portable devices in ophthalmology

**Authors:** Fernando Korn Malerbi, Paulo Henrique Ávila Morales, Caio Vinicius Saito Regatieri

**Affiliations:** 1 Department of Ophthalmology, Universidade Federal de São Paulo, São Paulo, SP, Brazil; 2 Instituto da Visão, São Paulo, SP, Brazil; 3 Tufts Medical School, Boston, Massachusetts, USA

Dear Editor,

We greatly appreciate the valuable input by Dr. Daniel A. Ferraz and colleagues on our recently published editorial^(1)^. We agree with their conclusion on the great potential of portable devices to close the access gaps in eye care, especially among the vulnerable and underserved populations.

Interestingly, we recently examined diabetic retinopathy (DR) screening models based on portable retinal cameras, both in the urban^(2)^ and remote, rural settings^(3)^; in both these scenarios, a strategy based on telemedicine and portable, low-cost retinal cameras is believed to increase the coverage of eye examinations.

We strongly believe that telemedicine, portable retinal cameras, and artificial intelligence are strong tools that, when well-coordinated, can reduce the cases of preventable blindness in low-to-middle income countries, as with the successful screening programs conducted elsewhere^(4)^. The design of the Brazil’s Family Health Strategy is compatible with the capillarization of such actions toward health care.
